# Strategies to Mitigate the Adverse Impacts of Viral Infections on Honey Bee (*Apis mellifera* L.) Colonies

**DOI:** 10.3390/insects16050509

**Published:** 2025-05-10

**Authors:** Ivana Tlak Gajger, Hossam F. Abou-Shaara, Maja Ivana Smodiš Škerl

**Affiliations:** 1 Department of Biology and Pathology of Bees and Fish, Faculty of Veterinary Medicine, University of Zagreb, Heinzelova 55, 10000 Zagreb, Croatia; 2 Department of Plant Protection, Faculty of Agriculture, Damanhour University, Damanhour 22516, Egypt; hossam.farag@agr.dmu.edu.eg; 3 Agricultural Institute of Slovenia, Hacquetova ulica 17, 1000 Ljubljana, Slovenia

**Keywords:** viruses, honey bees, *Varroa destructor*, stressors, genetics, pollination

## Abstract

Honey bees (*Apis mellifera*) play a crucial role in global food production through the pollination of various crops. These vital insects are susceptible to a range of viral pathogens that can disrupt their normal behavior and physiology, ultimately affecting colony dynamics and survival. There are diverse viruses that infect honey bees at different life stages, with a year-round prevalence. There are multiple pathways through which viruses can be transmitted among colonies. Notably, there is also a lack of commercial treatments against viral infections in bees, but some promising strategies exist to mitigate their negative effects, including vector control, and the implementation of good beekeeping practices and biosecurity measures. While methods for treating infected colonies have garnered attention, they receive less focus compared to aspects like transmission methods and seasonal prevalence of viruses. This article aims to review the aforementioned strategies in light of the available literature. It presents succinct and practical approaches categorized based on their potential direct or indirect effects on viruses, providing beekeepers and researchers with an overview of both fully established and still-developing methods. Controlling the ectoparasitic *Varroa destructor* mite population, which significantly impacts viral prevalence and virulence in bees, is crucial for reducing infections. Practical approaches such as selectively breeding honey bee populations resistant to viruses and ensuring proper nutrition are important strategies. Moreover, genetic methods have also been proposed and tested. The article not only emphasizes these methods but also discusses knowledge gaps and suggests future solutions to improve the health and productivity of honey bee colonies.

## Simple Summary

Honey bees (*Apis mellifera*) are essential for global food production through pollination, but are highly vulnerable to viral infections that threaten their strength and survival. Honey bee viruses spread year-round through various transmission pathways, with no available commercial treatments. However, strategies such as improved beekeeping practices, selectively breeding virus-resistant bee populations, hyperthermia treatment, and biosecurity measures can help mitigate their impacts. Controlling *Varroa destructor* mite, a major vector of honey bee viruses, is an important and necessary strategy. RNA interference (RNAi) is a promising approach for combating honey bee viruses. This review highlights potential solutions, identifies knowledge gaps, and suggests ways to enhance honey bee colony health.

## 1. Introduction

Global food production significantly depends on insect pollinators, especially economically and ecologically important honey bee (*Apis mellifera*) colonies [1,2,3,4]. Beekeeping is considered crucial to the agricultural sector, with the number of managed honey bee colonies steadily increasing, as reported from 1961 to 2017 [5]. However, the reported loss in honey bee colony numbers at various geographical locations poses a significant threat to global food security and biodiversity [6,7]. One example of loss in honey bee colonies involves colony collapse disorder (CCD), which raised concerns in the USA in 2006 and 2007 [8,9]. Various biotic and abiotic factors play a role in the loss of honey bee colonies [10], making it challenging to pinpoint a single stressor as the primary cause in most cases. However, viruses have emerged as a significant focus and have been identified as one of the devastating factors leading to colony losses [11,12,13].

Many viruses—at least 18—have been identified in honey bees [14,15]. Some viruses specifically target immature stages, while others can infect both immature and mature bees [15]. Typically, the names of economically important viruses indicate the major characteristic clinical signs they cause. For instance, sacbrood virus (SBV) induces changes in the unsealed brood, black queen cell virus (BQCV) targets developing bee queens, deformed wing virus (DWV) leads to wing deformities in newly emerged bees, and acute bee paralysis virus (ABPV) induces paralysis in adult bees [14]. These viruses have a global prevalence, with some, like DWV, exhibiting multiple genotypes (as reviewed in [16]); for additional information, refer to studies such as [17,18,19]. Many times, honey bee colonies are infected with multiple viruses [20,21,22]. Such concurrent infections can significantly impact the development and survival of honey bee colonies [22,23].

The significant impact of viruses on honey bees becomes apparent when considering the diverse transmission pathways involved [24]. Some viruses can spread from drones to queens during mating [25,26], and from queens to their offspring during egg-laying [27]. Transmission can also occur from worker bee to worker bee and between other castes of the same generation through direct contact in the hive [28]. Honey bee behaviors such as drifting and robbing are responsible for virus transmissions between hives and apiaries, and contaminated hive products also can contribute to higher virus prevalence [29]. Furthermore, viruses can be transferred among various species of insect pollinators as they visit flowers [30,31,32]. *Varroa destructor* mites are known to significantly increase virulence and facilitate the spread of viruses among honey bees [33]. Additionally, some honey bee viruses have been detected in various pests such as ants, hornets, and small hive beetles [34,35,36]. Transmission and spreading of viruses over long geographical distances is possible due to the transport and global trade of live honey bee stocks, honey bee products, and beekeeping equipment [37].

Infections with viruses can be detected year-round, although the prevalence of a specific virus may exhibit seasonal variations depending on the geographical location. For instance, BQCV prevalence in Greece is higher during the summer months [38], while SBV prevalence peaks in Southwest Germany during winter [39]. In most cases, viruses can weaken honey bee colonies without directly causing their demise [12]. Regrettably, there are no commercially registered treatments against diseases caused by honey bee viruses. Nonetheless, certain beekeeping practices and ongoing research efforts hold promise in alleviating the adverse impacts of viruses on honey bee colonies. These aspects are the focal points of this review, as elaborated in the following paragraphs, which present succinct and practical approaches rather than extensive reviews that were covered in previously published articles. To facilitate the presentation of the key points in the article, the proposed strategies were categorized based on their potential direct or indirect effects on viruses.

## 2. Strategies with Potential Indirect Effects on Viruses

Implementing these strategies is anticipated to indirectly mitigate viral infections by decreasing the infestation levels of *V. destructor* mites (a major vector of honey bee viruses) or enhancing overall honey bee health. Concurrently, these strategies may have limited direct impact on virus infectivity, virulence, or viral loads.

### 2.1. Varroa Control

The *V. destructor* mite plays a pivotal role in the spread of viruses within honey bee populations, colonies, and apiaries. These mites can transfer between forager worker bees during flower visits [40,41] and transmit viruses as they feed primarily on honey bee fat body tissue [42]. Numerous studies have highlighted the significant contribution of *V. destructor* to viral prevalence among honey bees, such as with the Kashmir bee virus [43]. *Varroa destructor* can damage honey bee colonies, severely impacting their development and productivity [44]. Bees exposed to high infestation levels of *V. destructor* during their development often exhibit lower protein levels and compromised immunity [45,46]. Thus, the timely and appropriate control of Varroosis is crucial for ensuring the health and survival of honey bee colonies [47], and it aids in reducing viral loads and the likelihood of virus transmission between colonies. Fortunately, a range of biotechnical measures and control methods for reducing the number of *V. destructor* mites exist, including mechanical interventions, treating with natural compounds, organic acids, and acaricidal medicines [47,48,49,50,51,52]. Beekeepers should diligently monitor *V. destructor* mite infestation levels in honey bee colonies using methods like powdered sugar [53] and promptly treat against *V. destructor* mites when their number is above the threshold level. As an example, such a threshold level has been estimated to be <1% mite infestation in August (the fall) under Canadian conditions [54]. This proactive approach not only helps with Varroosis control but also plays a crucial role in mitigating viral infections [51] and can be viewed as a fundamental strategy for reducing viral infections in honey bee colonies.

### 2.2. Queen Quality

In natural conditions, each honey bee colony typically has a single queen, and all offspring within the colony inherit its genetic traits [55]. The quality of queens is influenced by various abiotic and biotic factors, including rearing conditions and pathogen infections [56,57]. The mating success of queens relies on the number and quality of drones they mate with [58,59]. Some viruses can be transmitted to queens through infected drone semen or other ways, leading to vertical transmission (transovarian or transovum) during egg laying. Thus, some viruses, such as DWV and SBV, can be detected in eggs [27,60]. Requeening, or replacing queens, is crucial in honey bee colonies with high levels of brood virus infections or mortalities to prevent potential vertical virus transmission to offspring. Replacing old or underperforming queens with younger, healthier ones is a key strategy to protect honey bee colonies from collapse. Honey bee colonies led by young, selected queens exhibit better resistance to *V. destructor* mites and higher overall performance compared to those with older queens [61]. Promptly requeening colonies with unhealthy or aging queens is essential for ensuring their survival and development; however, this critical step may sometimes be overlooked by beekeepers.

### 2.3. Nutrition

Honey bees rely on nectar as an energy source and pollen as a vital source of essential amino acids and proteins [62,63]. The relationship between nutrition and immunity is well-established [64], with pollen playing a significant role in honey bee immunity by supplying essential amino acids [65,66]. Nectar and honey are essential for metabolic processes, with components that vary among different flowering resources, some of which possess antimicrobial properties [67]. While honey bee colonies are naturally fed on nectar and pollen during active seasons, the availability of these natural sources does not always ensure the provision of all necessary nutrients. Areas with limited pollen diversity may not be optimal for honey bee health [68]. To ensure bees receive an adequate and diverse diet, it is crucial to select areas with a wide variety of flowering plants. During dearth periods, such as late autumn and winter, natural food sources become scarce. Beekeepers should supplement their honey bee colonies with suitable food alternatives (substitutes) according to good beekeeping, environmental, and veterinary practices.

Extensive research has been conducted on alternative food sources for honey bees, with studies focusing on sugar feeding [69,70] as well as protein feeding [71,72]. Beekeepers must provide their colonies with appropriate nutrition to prevent starvation and enhance their overall health. While pollen and nectar are optimal for gene expression related to immunity and detoxification [73,74], supplements can also be beneficial for boosting honey bee immunity. These supplements may include vitamin C or plant extracts [75]. Notably, certain plant extracts like cinnamon, mint, and chamomile have been shown to enhance bees’ food consumption and pathogen-fighting abilities [55]. Furthermore, ethanolic propolis extract and honey bee venom have demonstrated antiviral potential in laboratory settings and could serve as dietary supplements for honey bees [76]. Honey bees with robust immunity are expected to better withstand viral infections compared to those with weaker immune responses [77]. Consequently, further research is recommended on ideal food additives, particularly plant-derived extracts, to enhance bee immunity in situations where artificial feeding is necessary or in regions with limited floral diversity [72,74,78].

### 2.4. Apiary Location

The location of an apiary can significantly influence the prevalence of viruses, a phenomenon that can be attributed to several factors: (1) the density of apiaries in the area (i.e., crowding), (2) the diversity of wild bee populations nearby, (3) the availability of suitable bee forage, and (4) environmental health. High honey bee colony density in a specific area can promote *V. destructor* mite infestations among colonies, and reinfestations between apiaries [79,80], leading to subsequent viral infections. Viral spillover between wild bees and honey bee colonies is possible [32,81,82], often linked to the presence of diverse wild bee populations near to apiary location [83]. As discussed in the nutrition section, inadequate bee foraging can hinder honey bee development and immunity, weakening their ability to combat viruses. The prevalence of viruses is influenced by environmental factors, particularly temperature, which may explain why certain locations exhibit higher rates of honey bee viruses [11]. The prevalence of other honey bee pathogens and *V. destructor* mites can also be influenced by environmental factors [84]. When selecting a permanent location for the apiary, choosing a suitable site with a low density of apiaries, availability of suitable bee forage, and without environmental stresses such as extreme heat stress is crucial and can help mitigate the negative impact of viruses. Moving colonies from their permanent location to another suitable site is essential when virus prevalence persists despite implementing the aforementioned strategies, necessitating the relocation of the apiary. Regarding migratory beekeeping activities, when moving colonies during blossom periods, selecting the temporary site should be performed carefully, especially in terms of a low density of apiaries. At present, there are no established rules that set standards regarding the suitable distances between apiaries or the appropriate number of colonies in a given apiary, especially considering that certain apiaries specialize in specific activities such as targeted pollination, queen or royal jelly production, or bee venom production alongside honey production. This gap presents an opportunity for future research.

## 3. Strategies with Potential Direct Effects on Viruses

When implementing these strategies, it is anticipated that viral infections will be reduced through direct effects on virus infectivity or viral loads. Meanwhile, these strategies can also exert indirect effects on viral infections by controlling *V. destructor* mites.

### 3.1. Honey Bee Stock

There are numerous subspecies of honey bees, estimated to be around 24 or more [85], each distinguished by unique morphological, behavioral, and genetic characteristics [86,87]. Hybridization and genetic enhancement have significantly contributed to the emergence of several hybrid honey bee varieties [55,88]. Certain honey bee stocks exhibit a natural resistance to *V. destructor* mites [89,90], notably those possessing Varroa Sensitive Hygiene (VSH) traits [91] or displaying a high inclination towards grooming behavior [92]. Honey bees with the capacity to combat *V. destructor* infestations also tend to have lower rates of clinical signs of viral infections within their colonies [93]. Reducing the influence of *V. destructor* mites on viral prevalence is anticipated to decrease, and consequently, the strategic selection of honey bee stocks capable of resisting mites plays a fundamental role in sustainably mitigating viral infections in colonies. Additionally, honey bee populations show varying levels of resistance to viral infections [94,95], indicating the possibility of selectively breeding viral-resistant bee populations in certain geographic regions. In support of this, American honey bee stocks (Minnesota Hygienic, POL-line, Russian bees, Italians, and Carniolans) have shown the presence of viruses in their queens without clear signs of infections; however, virus susceptibility varied among and within these stocks [94]. Despite no specific honey bee stock having a known ability to resist viral infections, selective breeding can still be conducted by beekeepers and queen breeders among their colonies to implement a sustainable strategy aiming to select colonies with low infections as sources for breeding queens for their colonies.

### 3.2. Thermal Treatment

Exposing honey bees to heat stress has demonstrated antiviral potential [96,97]. Consequently, subjecting honey bee colonies to hyperthermia could serve as a promising approach to alleviate the adverse impacts of viruses on honey bees [98]. The practice of exposing worker bees to hyperthermia, typically at temperatures around 40 to 42 °C for two to three hours, has been explored as a potential control method against *V. destructor* mites [99,100,101]. Several devices are available for thermally treating colonies, including the mite zapper [102], the thermovar [103], the thermo-solar hive [104], the Bee Ethic system [100], and the Vatorex [101]. Research has indicated that hyperthermia has the potential to reduce viral infections (specifically DWV and ABPV viral loads) in treated honey bee colonies using the Bee Ethic system [100]. This reduction may be attributed to hyperthermia’s role in decreasing *V. destructor* mite infestations within colonies, as well as its direct antiviral effects [98], particularly through the upregulation of heat shock proteins, which are components of the anti-viral immune pathways in honey bees. To avoid any potential harmful effects of inappropriate hyperthermia treatment, especially due to prolonged treatment exposure or elevated temperatures, particularly on immature stages, it has been suggested that treatment should be applied when brood-rearing activity notably declines, such as during the autumn period [98]. Given the availability of this method on a commercial scale, further studies are warranted to confirm its efficacy as a dual treatment against Varroosis and viruses.

### 3.3. RNA Interference

RNA interference (RNAi), a primary antiviral defense mechanism, operates through post-transcriptional gene silencing with sequence specificity, induced by double-stranded RNA (dsRNA) [105,106,107]. RNAi is a widely tested method for combating honey bee viruses. All technical aspects related to RNAi in honey bee viruses have been reviewed in [105], with key points highlighted in this article. The effectiveness of RNAi can be influenced by various factors, including the method of dsRNA delivery, which can be administered orally or via injection [108,109]. The mortality of treated worker bees may be associated with the chosen delivery method [110,111]. Generally, oral delivery is deemed more practical for colony-wide application, although further research in this area is warranted. Within a honey bee colony, bees go through various life stages (egg, larvae, pupae, adults) with differing ages, developmental statuses, and activities [112]. This intricate social structure of honey bees can pose a challenge that may affect dsRNA delivery to specific developmental stages and its efficacy. Honey bee colonies can harbor multiple viruses and pathogens at the same time, complicating the effectiveness of RNA interference (RNAi) and increasing the risk of off-target effects [113,114,115]. A significant challenge with RNAi is its cost, particularly since treatments often require repeated administration to each honey bee colony. Moreover, the rapid degradation of dsRNA poses concerns. An emerging approach involves utilizing the engineered edible photosynthetic cyanobacterium *Synechococcus elongatus* UTEX 2973 to stimulate RNAi immune responses in honey bees, with a primary focus on combating the DWV [116]. While RNAi holds promise as a potential solution for honey bee viruses, its implementation necessitates thorough study and evaluation.

### 3.4. Disinfection

The disinfection of beekeeping tools is a crucial practice for reducing the prevalence of viruses and pathogens within honey bee colonies. This process is typically carried out at the end of the beekeeping season or before its commencement. Beekeepers employ various methods such as subjecting beehive boxes, frames, and other tools to temperature extremes (e.g., blow torch flames, boiling in water solution of natrium hydroxide), disinfectants, or irradiation [117,118,119,120]. This practice is largely guided by beekeepers’ experience, given the absence of standardized protocols for disinfecting beekeeping tools. Some commercial disinfectants are available for use with small equipment like queen rearing tools and hive tools. While exposing wax combs to different temperatures (−20 °C, 5 °C, and 20 °C) over a month has not shown a significant reduction in virus levels, such as DWV, irradiation has demonstrated potential [121]. Therefore, the development of specific disinfectants, irradiation methods, or heat treatments for effectively and economically eliminating virus particles from beekeeping tools appears to be a critical area for further research. Furthermore, the recycling of old beeswax and the utilization of clean wax sheets are vital beekeeping, veterinary, and environmental good practices for safeguarding the health of honey bee colonies [118].

## 4. Outlooks

**Figure 1:**
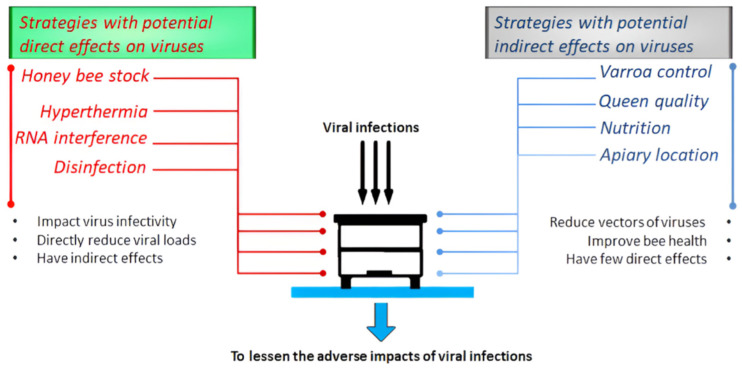
Strategies that can be used to mitigate the negative effects of viral infections in bee colonies, categorized into their potential direct or indirect effects on viruses.

A variety of measures can be adopted by beekeepers to safeguard honey bee colonies from the harmful effects of viruses and their transmission. Choosing an appropriate apiary location, selectively breeding viral-resistant bee populations, maintaining young and high-quality queens in colonies, regularly and appropriately treating against Varroosis, providing colonies with adequate additional diets [122], and utilizing heat treatments can be viewed as integrated strategies for virus control (Figure 1). Extensive research should be conducted in these areas to offer beekeepers user-friendly and cost-effective methods. Geographical factors must also be taken into consideration when devising integrated strategies against viral infections in honey bee colonies, particularly concerning the presence of different local ecotypes and honey bee subspecies, various additional feeding options, and diverse disease control methods. Although RNAi technology is not yet commercially accessible for beekeepers, technological advancements may facilitate the practical implementation of this antiviral defense mechanism in the future.

Due to the critical role of reproductive castes (drones and queens) in virus transmission through mating and egg-laying activities [27,60], it can be argued that focusing on strategies that exclusively target these reproductive castes can effectively safeguard entire honey bee colonies from the destructive impacts of viruses. Ensuring the presence of high-quality queens and drones free from viral infections or with minimal viral loads emerges as a key strategy for adoption by queen breeders. Encouraging queen breeders to adhere to standardized methods for queen rearing and evaluating queen quality is of significant importance [123]. Concurrently, in-depth research on the immunity of honey bee queens and drones should be prioritized to ensure the availability of reproductive castes with a heightened ability to withstand viral infections. Particularly, existing studies on queen and drone immunity remain limited, such as investigations into the effects of age [124], heat stress [125], and sperm viability [126]. For instance, a study revealed that drones from colonies exhibiting high hygienic behavior displayed elevated expression of the immune gene peptidoglycan recognition protein S2 (PGRP-S2) [127]. This strategic approach is not contingent upon a specific subspecies or honey bee stock but rather serves as a comprehensive technique involving the rearing, feeding, and management of reproductive honey bee castes.

An additional crucial strategy should focus on the drone congregation areas (DCAs), where aerial mating takes place [128]. These sites possess distinct characteristics and can be utilized by honey bees for many years [129,130]. It is imperative to identify and specify these areas, a task that can be accomplished through methods such as queen pheromones and geographical analysis [131,132]. By analyzing drone samples, these specific locations can be monitored to detect the presence of viruses or *V. destructor* mites [133,134]. Furthermore, queen breeders could be encouraged to use designated isolated DCAs to ensure that selected drones and queens mate exclusively. Given the crucial role of *V. destructor* mites in the spread of honey bee viruses, it is vital to consistently monitor and control parasitic mites, particularly in colonies intended for queen and drone rearing.

## 5. Conclusions

This article outlines various methods that can be implemented to mitigate the detrimental impact of viruses on honey bee colonies. There are several strategies that can be collectively employed to safeguard the overall health of honey bee populations. A fundamental and crucial step in virus management within honey bee colonies is the control of *V. destructor* mites. Alongside this, the recommended strategies include selectively breeding viral-resistant honey bee populations, utilizing young and high-quality queens, employing hyperthermia treatments, providing colonies with consistent year-round additional feeding, and incorporating genetically based approaches. Furthermore, relocating the apiary can be an additional strategy to consider under specific environmental and weather circumstances. As of now, there are no approved treatments for viral infections in honey bee colonies. The challenge lies in the potential simultaneous infection of honey bee colonies with multiple economically important viruses and the presence of various pathways for virus transmission among adult bees, colonies, and apiaries. Nevertheless, the strategies outlined in this article can serve as a foundation for developing comprehensive management plans to combat viral infections in honey bee colonies. Further studies are recommended to provide detailed explanations and quantitative data on how these mitigation strategies may reduce the number of viruses and/or the viral loads in managed honey bees, aiming to fill the current gaps in knowledge.

## Data Availability

No new data were created or analyzed in this study.
